# Peripheral Mononuclear Cell Resistin mRNA Expression Is Increased in Type 2 Diabetic Women

**DOI:** 10.1155/2008/892864

**Published:** 2008-12-21

**Authors:** Panayoula C. Tsiotra, Constantine Tsigos, Eleni Anastasiou, Eleni Yfanti, Eleni Boutati, Emmanouil Souvatzoglou, Ioannis Kyrou, Sotirios A. Raptis

**Affiliations:** ^1^Division of Basic Sciences, Hellenic National Center for the Research, Prevention and Treatment of Diabetes Mellitus and Its Complications (HNDC), 10675 Athens, Greece; ^2^Department of Internal Medicine, First Endocrine Section and Diabetes Centre, “Alexandra” Hospital, 11528 Athens, Greece; ^3^Second Department of Internal Medicine, Research Institute and Diabetes Center, Athens University Medical School, “Attikon” University General Hospital, Haidari, 11527 Athens, Greece

## Abstract

Resistin has been shown to cause insulin resistance and to impair glucose tolerance in rodents, but in humans its physiological role still remains elusive. The aim of this study was to examine whether resistin mRNA expression in human peripheral mononuclear cells (PBMCs) and its corresponding plasma levels are altered in type 2 diabetes. Resistin mRNA levels were easily detectable in human PBMC, and found to be higher in DM2 compared to healthy women (*P* = .05). Similarly, mononuclear mRNA levels of the proinflammatory cytokines IL-1*β*, TNF-*α*, and IL-6 were all significantly higher in DM2 compared to control women (*P* < .001). The corresponding plasma resistin levels were slightly, but not significantly, increased in DM2 women (*P* = .051), and overall, they correlated significantly with BMI (*r* = 0.406, *P* = .010) and waist circumference (*r* = 0.516, *P* = .003), but not with fasting insulin levels or HOMA-IR. Resistin mRNA expression is increased in PBMC from DM2 women, together with increased expression of the inflammatory cytokines IL-1*β*, TNF-*α*, and IL-6, independent of obesity. These results suggest that resistin and cytokines might contribute to the low-grade inflammation and the increased atherogenic risk observed in these patients.

## 1. INTRODUCTION

Resistin and other molecules commonly called adipokines, such as TNF-*α*, IL-6, and visfatin, are produced and secreted by both adipocytes and macrophages, and have been suggested to be important players in the pathogenesis of insulin resistance and atherosclerosis [1, 2]. Increased production of these adipokines occurs with expanding obesity, particularly visceral obesity, by both the adipocytes and the nonfat cells, mostly macrophages that infiltrate the adipose tissue [3, 4]. Indeed, the common origin of macrophages and adipocytes has been well documented as well as their overlapping biology and function (accumulation of lipids, expression and secretion of similar cytokines, such as TNF-*α*, IL-6, resistin, visfatin, and expression of genes involved in lipid metabolism) [4, 5]. Furthermore, recruitment and accumulation of macrophages in the adipose tissue are characteristics of obesity [6]. Interestingly, in humans, the expression of resistin and visfatin is predominantly detected in the macrophages populating the adipose tissue [7]. Several studies have also demonstrated increased expression and release of the proinflammatory cytokines, TNF-*α*, IL-1*β*, and IL-6, from the adipocytes or the macrophages infiltrating the adipose tissue [3, 7].

Notably, resistin belongs to a family of molecules called found in inflammatory zone (FIZZ), since a member of this family was found to be expressed in bronchial epithelial cells during allergic pulmonary inflammation in mice [8]. In human mononuclear cells, where resistin is highly expressed, its expression is markedly upregulated by lipopolysaccharide (LPS) and the proinflammatory cytokines (Il-6, TNF-*α*, and IL-1), acting via the NF-*κ*B-dependent pathway [9]. Furthermore, intravenous administration of endotoxin in humans markedly increased circulating resistin levels [9, 10]. In agreement with these experimental data, patients with severe inflammatory disease have significantly higher serum resistin concentrations, while patients with rheumatoid arthritis (RA) show increased resistin levels in their inflamed joints that correlate with markers of inflammation [11]. Furthermore, PPAR*γ* agonists and HMG-CoA reductase inhibitors (statins), both recognized for their anti-inflammatory properties, decrease macrophage resistin mRNA and protein secretion, apparently via the NF-*κ*B pathway [12, 13].

Consistent with the proinflammatory nature of resistin, human resistin powerfully stimulates TNF-*α*, IL-1*β*, IL-6, and IL-12 expression in human PBMC and murine macrophages, an effect that can be abrogated by an NF-*κ*B inhibitor, indicating the importance of this signalling pathway in the resistin-mediated inflammation [14]. Resistin was also shown to increase lipid accumulation in human macrophages [15]. Resistin also acts on endothelial cells and promotes their activation, by inducing the expression and release of monocyte chemoattractant protein (MCP)-1, endothelin-1 (ET-1), and adhesion molecules, such as vascular cell adhesion molecule -1 (VCAM-1) and intracellular adhesion molecule-1 (ICAM-1), while at the same time adiponectin abrogates this effect [16]. Moreover, resistin is secreted by the macrophages localizing to the atheromas, thereby promoting atherosclerosis in humans [17]. Indeed, patients with coronary artery disease (CAD) have higher serum resistin levels that correlate with markers of inflammation, and are predictive of coronary atherosclerosis in humans [18, 19]. High serum resistin levels have also been proposed as predictors of early atherosclerosis in obese children [20].

While resistin's role as an inflammatory marker in human's and rodent's physiology has been well documented, its role in obesity and insulin resistance in humans is still under debate. It is important that human resistin is expressed in much higher levels in macrophages than in adipocytes [21], quite the opposite from the case of mice, in which resistin clearly impairs insulin sensitivity [22, 23]. Several human studies have failed to confirm any relationship of circulating resistin levels with BMI, insulin sensitivity, or other metabolic parameters [24–26]. However, there have been reports of increased serum resistin levels in individuals with obesity and/or type 2 diabetes [27–29].

In the present study, we examined whether resistin TNF-*α*, IL-6, and IL-1*β* mRNA levels from human peripheral mononuclear cells are altered in type 2 diabetes and whether they correlate with circulating resistin levels and with indices of obesity or insulin resistance.

## 2. SUBJECTS AND METHODS

### 2.1. Subjects

The study was approved by the “Alexandra” Hospital Ethical Committee and all subjects gave informed consent. Forty eight (48) women, all premenopausal, aged between 21 and 49 years, participated in our study. Twenty two had type 2 diabetes (DM2), and twenty six were healthy controls with normal glucose tolerance (NGT). Both groups were further divided into two groups: those with BMI < 27 and those with BMI > 27 (Table 1). All women have had a stable weight, and they were on a normal eucaloric diet and normal physical activity, not participating in any specific exercise program for the last three months preceding the test. Of the DM2 women, 15 were on diet alone, 4 on oral hypoglycaemic agents, 2 on insulin, and 1 on combined insulin and oral hypoglycaemic agents. No DM2 or healthy woman was on thiazolidinedione or statin medication. All control subjects had no history of endocrine abnormality and were on no medications.

Body mass index (BMI) was calculated as the ratio of body weight (Kg) per height (m^2^). Insulin resistance was calculated by the homeostasis model assessment index (HOMA-IR) as [fasting insulin (*μ*U/mL) × fasting glucose (mmol/L)]/22.5. Insulin sensitivity was calculated by the Matsuda index as (10 000/sqare root of [fasting glucose × fasting insulin] × [mean glucose during OGTT × mean insulin during OGTT]). Fasting glucose, triglycerides, and free fatty acid (FFA) levels were analyzed on a Falcor 300 Chemical Analyser (Menarini Diagnostics, Italy) and fasting plasma insulin levels were measured by IRMA (INSI-CTK, DiaSorin, Italy), in all individuals. 

### 2.2. RNA preparation

Peripheral blood was collected from all women after an overnight fast and the mononuclear cells were separated using a Histopaque-1077 gradient (Sigma-Aldrich, USA). At the same time, plasma was kept at −80°C for subsequent measurement of adipokines.

The mononuclear cells were then left to adhere for 1 hour for the selective isolation of the primary monocyte-derived macrophages, and total RNA was extracted from cells as previously described [30]. Briefly, after the RNA isolation, cDNA synthesis was performed in all samples using oligo random primers (NEB, Mass, USA) and MMLV reverse transcriptase (Promega Corp., Wis, USA) [30]. We developed a quantitative real-time PCR using fluorescently labeled hybridization probes to detect resistin and proinflammatory (IL-1*β*, TNF-*α*, and IL-6) mRNA expression in human mononuclear cells, in a spectrofluorometric thermal cycler (LightCycler, ROCHE, Mannheim, Germany).

### 2.3. Primer design

Hybridization-specific primers and hybridization-specific probes for all genes were designed and manufactured by the (TIB MOLBIOL Berlin, Germany). Hybridization primers and probes for all target and reference genes were showen in Table 2.

Primers were chosen to lie between exons whenever possible and the analysis was performed basically with the program OLIGO 6.0 (Molecular Biology Insights, Cascade, Colo, USA).

### 2.4. Quantitative real-time PCR

Relative quantification is the method that determines the changes in steady-state mRNA levels of a gene across various samples and expresses them relative to the levels of an internal reference control gene, usually a housekeeping gene. In order to quantify resistin, IL-1*β*, TNF-*α*, and IL-6 relative mRNA levels, we used the calibrator—normalized standard curve and the LightCycler Relative Quantification 1.0.1 Software (Roche, Mannheim, Germany), as previously described [30]. Briefly, using this quantification method, results are expressed as the target/reference ratio of the sample divided by the target/reference ratio of the calibrator, and it does not require a standard curve in each run (Roche Applied Science, Technical Note No. LC 13/2001-Relative Quantification). The human housekeeping genes of *β*-actin and porphobilinogen deaminase (PBGD) were used as standards for normalization. Selection of either *β*-actin or PBGD as housekeeping genes was necessary because the copy number of the housekeeping gene should be in a similar range with that of the target gene to make comparative quantification possible (Roche Applied Science, Technical Note No. LC 15/2002-Selection of Housekeeping Genes). Thus, the PBGD house keeping gene was chosen for the relative quantification of the IL-6 mRNA expression because of the very low level of IL-6 expression in the calibrator (HL60 induced with phorbol myristyl acetate (PMA)) mRNA.

Standard curves describing the PCR efficiencies of the target (resistin, IL-1*β*, TNF-*α*, or IL-6) and the reference genes (*β*-actin or PBGD) were created from a dilution series of the calibrator cDNA, using the Second derivative maximum method with the arithmetic baseline adjustment. The second derivative maximum method was used for the determination of the various crossing points (Cps) of the target and the reference gene in each sample. Finally, a corresponding coefficient file was created, which determines the efficiency for the target and the reference gene. Amplification of each sample was performed in triplicate, in different runs, and fluorescence resulting from the close proximity of the annealed hybridization probes was detected at the end of the annealing step of each PCR cycle.

The results from different PCR runs were run randomly in ethidium bromide-stained agarose gels and photographed in an Image Analyser VDS System (Amersham-Pharmacia Biotech, Sweden).

**Immunoassays**

Circulating levels of resistin (BioVender, Canal zone, USA), TNF-*α* (R&D Systems, UK), and IL-6 (R&D Systems, UK) were measured in the plasma of all women, using standard commercial enzyme-linked immunosorbent assays according to the manufacturers'-recommended protocols. IL-1*β* circulating protein levels were measured in the plasma of all women, using a high sensitivity multiplex assay (xMAP technology) and the fluorescently labeled microsphere beads (Linco-Millipore Corp., USA), in a LUMINEX 200 instrument (Luminex Corp., USA). We could not detect IL-1*β* in the majority of our subjects (in 57% of controls and 55% of DM2), making the analysis of circulating IL-1*β* problematic.

The sensitivities of the assays were 0.2 ng/mL (resistin), 0.12 pg/mL (TNF-*α*), <0.094 pg/mL (IL-6), 0.06 pg/mL (IL-1*β*), the intra- and the interassay coefficients of variation were, respectively, 3.6% and 6.7% (resistin), 6.6% and 13.4% (TNF-*α*), 6.9% and 14.1% (IL-6), 3.11% and 2.16% (IL-1*β*). All samples were measured in duplicate.

### 2.5. Statistical analysis

Statistical analysis was performed using the SPSS version 14.0.1 software (Chicago, Ill, USA). Data are expressed as mean ± SEM of *n*-independent experiments. Nonparametric statistical analyses were applied (Spearman's correlation test and nonparametric Mann-Whitney test), where appropriate. A *P*-value of less than .05 was considered statistically significant.

## 3. RESULTS

### 3.1. Clinical characteristics of study subjects

Table 1 shows the clinical characteristics of the DM2 and healthy NGT women, who were further divided into two subgroups according to their BMI, one with BMI < 27 and one with BMI > 27. The groups were generally well matched between them. DM2 women, however, had significantly higher waist circumference, triglyceride and FFA levels, and lower HDL cholesterol levels compared to the BMI-matched controls.

Resistin, TNF-*α*, IL-6, IL-1*β* mRNA expression from human mononuclear-monocytic cells is elevated in DM2 women.

**Figure 1:**
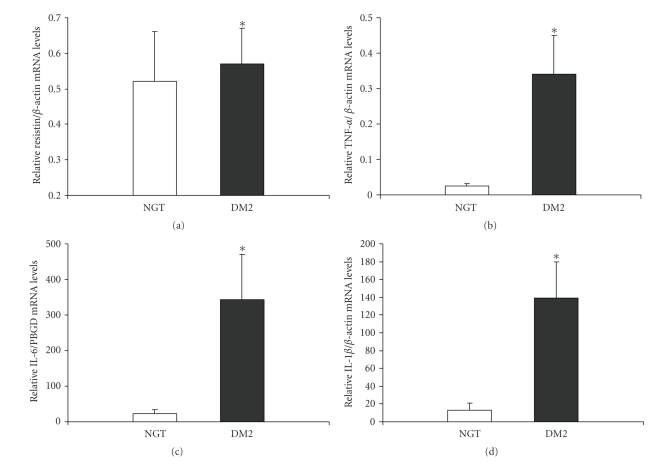
(a) Bar graphs compare relative resistin/*β*-actin mRNA levels, (b) TNF-*α*/*β*-actin mRNA levels, (c) IL-6/PBGD mRNA levels, and (d) IL-1*β*/*β*-actin mRNA levels, from peripheral mononuclear-enriched monocytes from NGT-control and DM2 women. *P < .05 versus NGT-women.

Resistin mRNA expression was easily detected in human mononuclear-monocytic cells in both DM2 and NGT women, and was significantly higher (0.57 ± 0.1 versus 0.52 ± 0.1 arbitrary units, *P* = .05) in the former group (Figure 1(a)), while no significant differences were observed between the two BMI subgroups (Table 3).

TNF-*α*, IL-6 mRNA and IL-1*β* mRNA levels were significantly higher (∼10-, 15-, and 10-fold, resp.) in DM2 women compared to NGT women [0.34 ± 0.1 versus 0.03 ± 0.01 (*P* < .001), 342.98 ± 127.9 versus 21.64 ± 12.6 (*P* < .001), and 139.36 ± 40.2 versus 13.21 ± 7.8 arbitrary units (*P* = .001), resp.] (Figures 1(b), 1(c), and 1(d)).

Resistin mRNA levels correlated positively with mononuclear IL-1*β*, and TNF-*α*, mRNA levels (*r* = 0.324, *P* < .05 and *r* = 0.447, *P* < .01, resp.). Furthermore, TNF-*α* and IL-6 mRNA levels correlated positively between them (*r* = 0.687, *P* < .001) and also each one of them with the IL-1*β* mRNA levels (*r* = 0.767, *P* < .001 and *r* = 0.765, *P* < .001, resp.), the 2-hour OGTT glucose levels (*r* = 0.462, *P* = .005 and *r* = 0.486, *P* = .004, resp.) and negatively with HDL cholesterol levels (*r* = −0.367, *P* < .02 and *r* = −0.372, *P* < .02, resp.), while IL-1*β* mRNA levels correlated positively with BMI (*r* = 0.374, *P* = .017), waist circumference (*r* = 0.414, *P* = .019), fasting glucose levels (*r* = 0.365, *P* = .022), HOMA-IR (*r* = 0.325, *P* = .043), glycosylated hemoglobin (*r* = 0.542, *P* = .001), FFA levels (*r* = 0.335, *P* = .043), and negatively with HDL cholesterol levels (*r* = −0.395, *P* = .015).

### 3.2. Circulating resistin, TNF-*α*, IL-6, and IL-1*β* protein levels

**Figure 2:**
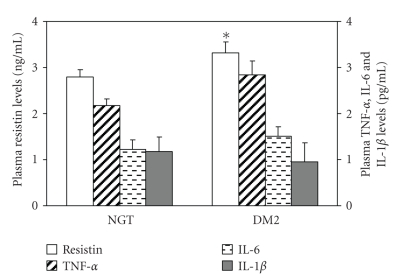
Comparison of circulating resistin, TNF-*α*, IL-6, and IL-1*β* protein levels between the NGT-healthy control and DM2 women. *P < .051 versus NGT-women.

Plasma resistin levels tended to be overall significantly higher in DM2 women compared to NGT control women (*P* = .051) (Figure 2), but no significant differences were observed between the two BMI subgroups. Circulating TNF-*α*, IL-6, and IL-1*β* protein levels did not differentiate between the DM2 and the NGT-control women (Figure 2), although in the subgroup of DM2 with BMI > 27, TNF-*α* and IL-6 plasma levels were significantly higher compared to NGT women (Table 4).

Circulating resistin correlated positively with mononuclear resistin, IL-6 and IL-1*β* mRNA expression (*r* = 0.526, *P* = .001 and *r* = 0.354, *P* = .029 and *r* = 0.425, *P* = .009, resp.), BMI and waist circumference (*r* = 0.406, *P* = .010 and *r* = 0.516, *P* = .003), but not with HOMA-IR, fasting insulin, or glucose plasma levels. TNF-*α* and IL-6 plasma levels also correlated with BMI and waist circumference (*r* = 0.361, *P* = .013 and *r* = 0.651, *P* < .001 and *r* = 0.379, *P* = .023 and *r* = 0.676, *P* < .001, resp.) as well as with fasting glucose, insulin and triglyceride levels, the HOMA-IR, while they correlated negatively with the Matsuda index and HDL cholesterol levels.

## 4. DISCUSSION

We have demonstrated that resistin mRNA expression from human peripheral monocyte-enriched mononuclear cells is elevated in type 2 diabetic women, compared to healthy control women, in parallel with significant increases in mononuclear IL-1*β*, TNF-*α*, and IL-6 mRNA expression.

Resistin, a 12.5-kDa cysteine-rich adipokine, which was shown to cause insulin resistance and to impair glucose tolerance in rodents [1], is also highly expressed in human mononuclear cells and macrophages [21]. While in rodents, adipocyte-produced resistin is mainly involved in glucose metabolism and insulin resistance: in humans, monocyte-produced resistin seems to have potent proinflammatory properties. Various inflammatory stimuli could result in hyperresistinemia in humans [9, 10], while human resistin can stimulate the expression of the inflammatory cytokines TNF-*α* and IL-6 in both human and murine macrophages, via an NF-*κ*B-dependent pathway [11, 14]. The proinflammatory nature of resistin was further documented by the findings that serum resistin concentrations were significantly elevated in patients with severe inflammatory disease and in patients with previous myocardial infarction, in whom they positively correlated with markers of inflammation, independent of CRP [11, 18–20].

In the present study, we have demonstrated that resistin mRNA levels from human peripheral monocyte-enriched mononuclear cells were higher in the DM2 compared to healthy women, which was independent of BMI. It should be noted that the difference did not reach significance between the individuals groups, but only when all patients were grouped together in NGT and DM2. This is obviously due to the small number of individuals used in our study, which is a weakness of our data. The above overall difference may suggest that, unlike inflammatory cytokine mRNA expression from human mononuclear cells, mononuclear resistin mRNA is not appreciably increased in vivo with chronic hyperinsulinemia that characterizes obesity. In support of this, in vitro studies demonstrated that insulin decreases resistin mRNA from human monocytes [31], while in the 3T3-L1 adipocytes, insulin downregulated resistin mRNA and protein secretion—possibly through the PI 3-kinase, ERK, or p38 MAP-kinase pathways—[32]. Recently, however, another study demonstrated that acute hyperinsulinemia is associated with increased resistin mRNA expression in human subcutaneous adipose tissue and a similar increase in plasma resistin levels [33].

Macrophages and adipocytes share an overlapping biology and function [4], and infiltration of the adipose tissue with the macrophages is characteristic of obesity [6]. Several recent studies suggest that obesity is associated with an increase in adipose tissue macrophages, which also participate in the inflammatory process through the elaboration of cytokines [3, 4, 34]. Proinflammatory cytokines (TNF-*α* and IL-6) and adipokines (resistin and visfatin), produced by the peripheral macrophages, can also be expressed in increased amounts in the fat tissue of obese individuals acting in parallel as important regulators of inflammation, atherosclerosis, and insulin sensitivity in both tissues. In a recent study, the expression of two adipokines, resistin, and visfatin was detected predominantly in the macrophages populating the human visceral fat tissue, suggesting that these adipokines might have an important role in the inflammatory load of obesity [7].

The putative role of resistin in obesity-induced insulin resistance was confirmed by studies, in rodents, demonstrating that administration of recombinant resistin impairs hepatic insulin sensitivity and glucose metabolism [22], while possibly playing a role in maintaining fasting blood glucose levels [23]. This was debated by various human studies demonstrating that there is no relationship of circulating resistin levels with BMI, insulin sensitivity, or other metabolic parameters [24–26]. However, other studies found increased serum resistin levels in individuals with obesity and/or type 2 diabetes [27–29]. The above conflicting data lead to the ascertainment that despite the initial belief that resistin could be the factor linking obesity and type 2 diabetes, its biologic significance in the pathogenesis of insulin resistance, at least in humans, is under controversy [35]. In fact, while in rodents adipose tissue is the only source of resistin production, in humans resistin is barely detectable in adipocytes, and actual resistin mRNA levels are much higher in monocytes and macrophages than in adipocytes [21].

In the present study, we have additionally demonstrated that plasma resistin levels are marginally increased in type 2 diabetic women compared to healthy women, and this increase was detected in both subgroups, those with BMI < 27 and those with BMI > 27. Thus, our data are inline with previous reports that found elevated serum resistin levels in type 2 diabetic subjects compared with nondiabetic subjects [28, 29, 36]. Nevertheless, we observed no correlation of circulating resistin with insulin resistance indices. However, the observed positive correlation of circulating resistin with BMI and waist circumference might support some association of resistin with obesity-induced insulin resistance. Therefore, the observed increased plasma resistin levels in the DM2 women could be due to the increased visceral obesity, as it reveals the higher waist circumference of these patients. Circulating Il-1*β* did not differentiate between the healthy control and the DM2 women, a result that lines with previous studies in which Il-1*β* alone cannot associate with the risk of developing type 2 diabetes, while Il-1*β* was even decreased in an impaired glucose tolerance (IGT) group compared to NGT subjects [37, 38]. It should be emphasized that due to the large number of subjects in whom plasma IL-1*β* levels were undetectable, they should be interpreted with caution. TNF-*α* and IL-6 plasma levels were also similar between the NGT and the DM2 women of our study, although their levels are significantly increased in the subgroup of DM2 women with BMI > 27, compared to NGT with BMI < 27 women; moreover, they were significantly correlated with the obesity and insulin resistance indices. While in the EPIC-Potsdam study the elevated levels of TNF-*α* and IL-6 were found to be strongly associated with future type 2 diabetes, other studies failed to demonstrate increased TNF-*α* and IL-6 plasma levels in DM2 patients [37, 39]. The above discrepancy could be due to the small number of subjects used in the later study, as it was also in our case, and due to ethnic differences between the various studies.

Obesity-induced insulin resistance is characterized by a chronic, systemic low-grade state of inflammation and is strongly associated with inflammatory markers, while inflammation may contribute to insulin resistance and atherosclerosis [2, 5, 40]. Therefore, with expanding obesity and increased macrophage infiltration of the adipose tissue, there is an increased cytokine production by both cell populations, with all the detrimental metabolic consequences on insulin resistance and on the inflammatory status [4, 6, 41]. In the present study, we have demonstrated that in type 2 diabetes, there is an increased mRNA expression of resistin, along with significant increases in TNF-*α*, IL-6, and Il-1*β* mRNA expression by the peripheral circulating mononuclear cells. The results from this study, together with previous data, showing higher mononuclear visfatin mRNA expression in the DM2 women [30], suggest that an overload of mRNA expression of inflammatory cytokines and adipokines occurs in the mononuclear cells from type 2 diabetic patients, aggravating the proinflammatory status of this condition. These data further support the notion that the peripheral circulating mononuclear cells can indeed be an important source of resistin production in humans. Thus, in type 2 diabetes, macrophages that infiltrate the stroma of the adipose tissue and/or the vascular endothelium can locally produce and secrete increased resistin and cytokine (Il-1*β*, TNF-*α*, and IL-6) levels, which may enhance the inflammatory load and the associated insulin resistance and vascular dysfunction observed in these patients. 

## Figures and Tables

**Table 1:** Patients' characteristics, their fasting glucose, fasting insulin, triglycerides, HDL and FFA levels, the HOMA-IR and Matsuda indices.

		*n*	BMI (Kg/m^2^)	Waist (cm)	Fasting glucose (mg/dL)	Fasting insulin (*μ*U/mL)	HOMA-IR	Matsuda	Triglycerides (mg/dL)	HDL (mg/dL)	FFA (mmol/L)
NGT	BMI < 27	14	22.5 ± 0.7	76.5 ± 2.3	87.5 ± 3.6	6.4 ± 0.6	1.4 ± 0.1	9.3 ± 0.9	63.2 ± 5.0	46.5 ± 3.4	0.43 ± 0.05
	BMI > 27	12	32.9 ± 1.5*	95.7 ± 3.0*	98.3 ± 2.1*	12.7 ± 2.1*	3.0 ± 0.5*	5.0 ± 1.3*	100.2 ± 18.9	44.0 ± 3.3	0.47 ± 0.05

DM2	BMI < 27	8	23.7 ± 0.7^§^	78.2 ± 3.4^§^	156.9 ± 32.9*^§^	8.3 ± 1.9	4.2 ± 2.1	6.3 ± 1.4	80.3 ± 17.4	39.0 ± 2.9	0.55 ± 0.10
	BMI > 27	14	36.4 ± 1.4*	108.1 ± 3.0*^§^	136.7 ± 13.0*^§^	16.6 ± 2.6*	5.6 ± 1.0*	2.4 ± 0.3*^§^	150.8 ± 26.5*	27.4 ± 1.5*^§^	0.59 ± 0.04*

Mean ± SEM: *P < .03 versus NGT-BMI < 27; ^§^ *P* < .05 versus NGT-BMI > 27.

**Table 2:** 

	5′-sense primer-3′	5′-a-sense primer-3′
Resistin	GGGCTGTTGGTGTCTAGCAAG	GTCTCGGCGCGCACAT
IL-1*β*	CAGGGACAGGATATGGAGCAA	GCAGACTCAAATTCCAGCTTGTTA
TNF-*α*	ACAAGCCTGTAGCCATGTT	AAAGTAGACCTGCCCAGACT
IL-6	CCAATCTGGATTCAATGAGGAGACT	GAGCCCTCAGGCTGGACTG
*β*-actin	CTTCTACAATGAGCTGCGTGTG	GTGAGGATCTTCATGAGGTAGTCAGTC
PBGD	GGCTGCAACGGCGGAA	CCTGTGGTGGACATAGCAATGATT

	5′-FL probe-3′	5′-LC Red640/LCRed705 probe-3′

Resistin	GCGACCTCCTGGATCCTCTCATTGA	GCTTCTTCCATGGAGCACAGGGTC
IL-1*β*	GCTTATCATCTTTCAACACGCAGGACA	GTACAGATTCTTTTCCTTGAGGCCCA
TNF-*α*	GCATTGGCCCGGCGGTTC	CCACTGGAGCTGCCCCTCAGCT
IL-6	AGATGCAATAACCACCCCTGACCCAA	CACAAATGCCAGCCTGCTGACGAA
*β*-actin	GGTATGCCCTCCCCCATGCC	TCCTGCGTCTGGACCTGGCTG
PBGD	CATACAGACGGACAGTGTGGTGGCAAC	TGAAAGCCTCGTACCCTGGCCTG

**Table 3:** Relative resistin, TNF-*α*, IL-6, and IL-1*β* mRNA levels from mononuclear cells between the subgroups of the NGT- and the DM2- women.

		Resistin/*β*-actin	TNF-*α*/*β*-actin	IL-6/PBGD	IL-1*β*/*β*-actin
NGT	BMI < 27	0.50 ± 0.14	0.03 ± 0.008	20.1 ± 16.1	5.4 ± 1.7
	BMI > 27	0.54 ± 0.26	0.03 ± 0.01	23.5 ± 20.7	23.1 ± 17.6

DM2	BMI < 27	0.70 ± 0.25	0.31 ± 0.16*^§^	242.0 ± 120.5*^§^	79.9 ± 44.0
	BMI > 27	0.48 ± 0.07	0.37 ± 0.16*^§^	405.1 ± 194.9*^§^	169.1 ± 55.1*^§^

Data expressed as mean ± SE: *P < .017 versus NGT-(BMI < 27)-women; ^§^ *P* < .013 versus NGT-(BMI > 27)-women.

**Table 4:** Circulating resistin, TNF-*α*, IL-6, and IL-1*β* protein levels between the subgroups of the NGT- and the DM2-women.

		Resistin (ng/mL)	TNF-*α* (pg/mL)	IL-6 (pg/mL)	IL-1*β* (pg/mL)
NGT	BMI < 27	2.65 ± 0.19	2.07 ± 0.19	0.80 ± 0.14	1.37 ± 0.39
	BMI > 27	2.98 ± 0.23	2.32 ± 0.20	1.77 ± 0.36*	0.97 ± 0.52

DM2	BMI < 27	3.24 ± 0.45	2.14 ± 0.45	0.93 ± 0.18^§^	1.19 ± 0.85
	BMI > 27	3.36 ± 0.29	3.24 ± 0.36*	1.84 ± 0.28*	0.79 ± 0.42

Data expressed as mean ± SE: *P < .015 versus NGT-(BMI < 27)-women; ^§^ *P* < .05 versus NGT-(BMI > 27)-women.
